# The Effect of Carbon Doping on the Crystal Structure and Electrical Properties of Sb_2_Te_3_

**DOI:** 10.3390/nano13040671

**Published:** 2023-02-09

**Authors:** Jie Zhang, Ningning Rong, Peng Xu, Yuchen Xiao, Aijiang Lu, Wenxiong Song, Sannian Song, Zhitang Song, Yongcheng Liang, Liangcai Wu

**Affiliations:** 1 College of Science, Donghua University, Shanghai 201620, China; 2 State Key Laboratory of Functional Materials for Informatics, Shanghai Institute of Microsystem and Information Technology, Chinese Academy of Sciences, Shanghai 200050, China; 3 Shanghai Institute of Intelligent Electronics & Systems, Shanghai 200433, China

**Keywords:** phase change random access memory, C-doped Sb_2_Te_3_, density functional theory, formation energy, continuously adjustable resistance value

## Abstract

As a new generation of non-volatile memory, phase change random access memory (PCRAM) has the potential to fill the hierarchical gap between DRAM and NAND FLASH in computer storage. Sb_2_Te_3_, one of the candidate materials for high-speed PCRAM, has high crystallization speed and poor thermal stability. In this work, we investigated the effect of carbon doping on Sb_2_Te_3_. It was found that the FCC phase of C-doped Sb_2_Te_3_ appeared at 200 °C and began to transform into the HEX phase at 25 °C, which is different from the previous reports where no FCC phase was observed in C-Sb_2_Te_3_. Based on the experimental observation and first-principles density functional theory calculation, it is found that the formation energy of FCC-Sb_2_Te_3_ structure decreases gradually with the increase in C doping concentration. Moreover, doped C atoms tend to form C molecular clusters in sp^2^ hybridization at the grain boundary of Sb_2_Te_3_, which is similar to the layered structure of graphite. And after doping C atoms, the thermal stability of Sb_2_Te_3_ is improved. We have fabricated the PCRAM device cell array of a C-Sb_2_Te_3_ alloy, which has an operating speed of 5 ns, a high thermal stability (10-year data retention temperature 138.1 °C), a low device power consumption (0.57 pJ), a continuously adjustable resistance value, and a very low resistance drift coefficient.

## 1. Introduction

With the continuous development of an information society, people's demand for information storage and calculation continues to grow. Phase change random access memory (PCRAM) is greatly welcomed by researchers in industrial electronics, artificial intelligence, and other fields because of its simple process, high integration, non-volatility, multi-level storage, and other characteristics [1]. The non-volatile memory technology 3D-XPoint, developed by Intel and Micron and announced for the first time in August 2015, is a new type of non-volatile memory that can significantly reduce latency, so that more data can be stored near the central processing unit [2], and its essence is PCRAM. Intel claims that its speed and life span are 1000 times that of NAND Flash, its integration density is 10 times that of traditional memory, and its cost is half that of Dynamic Random Access Memory (DRAM) [3].

The key technology of PCRAM lies in phase change materials. PCRAM realizes data storage by using the resistance difference of the phase change material between the amorphous state and the crystalline state. The common Ge_2_Sb_2_Te_5_ (GST) phase change material is the most widely used material at present, but it has the disadvantages of slow speed (~50 ns) and poor thermal stability (~82 °C) [4,5], which cannot meet the requirements of high-speed and high thermal stability PCRAM, thus limiting its application in electronic devices. In order to improve the performance of PCRAM, it is key to find phase change materials with fast reversible phase change speed and high thermal stability. Zuliani et al. explored the region rich in Ge element in the GST ternary diagram, which made the thermal stability of Ge-rich GST meet the specifications of automobile application, but the programming speed loss was about one-third of GST [6]. Diaz et al. improved the bottom electrode contact and thermal stability (1h retention is 230 °C) by adding a GST buffer layer under Ge-rich GST [7]. The high thermal stability of a Ge-rich GST alloy is due to the formation of local tetrahedral Ge-Ge bonds, which leads to a more disordered structure; that is, it is less prone to crystallization, which increases the resistivity of crystalline GST and reduces the RESET power consumption of PCRAM [6]. However, the operation speed of Ge-rich GST is still not satisfied.

In recent years, Sb_2_Te_3_ had great application potential in thermoelectrics [8], optoelectronics [9], PCRAM, and neuromorphic applications [10]. Sb_2_Te_3_ has a fast reversible phase transition, which is due to its rich Te and vacancies in the face-centered cubic (FCC) phase [11]. Sb_2_Te_3_ is considered one of the most advantageous candidate materials for PCRAM, but its thermal stability is poor [12,13,14]. In order to improve thermal stability while maintaining the advantages of its high crystallization speed, doping is a feasible means to improve the thermal stability of Sb_2_Te_3_ [15]. Although some dopants can improve the thermal stability of amorphous Sb_2_Te_3_, they will cause undesirable phase separation during the erasing and writing of PCRAM, which will seriously limit the crystallization speed and deteriorate the device’s performance [16]. Dopant C is a relatively ideal dopant [17], and doping Sb_2_Te_3_ with element C can not only achieve the purpose of fast phase change, high thermal stability, and low power consumption of RESET [18], but also will not cause serious phase separation. Experimental results show that there is an FCC phase in C-doped Sb_2_Te_3_ (C-Sb_2_Te_3_). Combined with density functional theory, we found that the formation of an FCC-Sb_2_Te_3_ structure can gradually decrease with the increase in C doping concentration. The doped C atoms tend to form C molecular clusters in sp^2^ hybridization at the grain boundary of Sb_2_Te_3_. Furthermore, PCRAM device cells based on C-Sb_2_Te_3_ were fabricated, which had an operating speed of 5 ns, high thermal stability, a low device power consumption, a continuously adjustable resistance, and an extremely low resistance drift coefficient.

## 2. Calculations and Experimental Section

### 2.1. Density Functional Theory (DFT) Methods

In the calculation work, we constructed a 3 × 3 × 3 supercell model of an FCC-Sb_2_Te_3_, which contains 180 atoms (72 Sb atoms and 108 Te atoms), and randomly generated 36 Sb cation vacancies in the system. We also constructed an FCC-Sb_2_Te_3_ model with 160 atoms of Σ3 twin grain boundary, including 64 Sb atoms, 96 Te atoms, and 32 random Sb cation vacancies. The thickness of the vacuum layer between supercells is 10 Å.

We use the Vienna ab initio simulation package (VASP) for density functional theory calculation. We adopted the projector augmented wave (PAW) potentials for describing the ion–electron interaction, the generalized gradient approximation (GGA) of Perdew-Burke–Ernzerhof (PBE) for exchange–correlation interactions between electrons [15,19,20,21,22,23]. The calculated valence electrons include 2s^2^2p^2^ of C, 5s^2^5p^3^ of Sb, and 5s^2^5p^4^ of Te, and the plane wave cutoff energy is set to 550 eV. With Γ point as the origin, the Monkhorst–Pack method is used to generate 1 × 1 × 1 and 3 × 3 × 1 k-point grids, respectively, and the Gaussian smearing method is used to adjust each orbital occupation. The cutoff energy and k-point grids have been tested, and the atomic structures of these two models are fully optimized. The energy convergence criterion is 10^−5^ eV, while the atomic force is less than 0.05 eV/Å. We implemented the Crystal Orbital Hamiltonian Population (COHP) bonding analyses using the LOBSTER setup [24].

### 2.2. Experimental Methods

By magnetron sputtering, the C and Sb_2_Te_3_ were co-sputtered onto a SiO_2_/Si (100) substrate, and the thickness of the film can be controlled by adjusting the sputtering time and sputtering power. The magnetron sputtering power of Sb_2_Te_3_ is 20W RF and the power of C is 40W DC. The deposition proceeds with Ar at a flow rate of 20 SCCM, with a background pressure of 3 × 10^−4^ Pa. The film was heated in situ at a heating rate of 60 °C/min in a self-made vacuum heating station, and the changes of resistance with time at various temperatures were recorded. Data retention for 10 years (and 100 years) was estimated by the Arrhenius Equation. To explore the electrical behavior of C-Sb_2_Te_3_ in memory cells, the PCRAM cells were fabricated with the traditional T-shaped (mushroom-type) structure. The bottom W heat electrode with a diameter of 190 nm was fabricated by 0.13 μm complementary metal oxide semiconductor technology. The bottom W heat electrode is covered with a C-Sb_2_Te_3_ film with a thickness of about 135 nm, and 40 nm TiN is deposited as the top electrode. The PCRAM cells were patterned using an etching process. The prototype PCRAM cells were annealed at 300 °C for 10 min in a N_2_ atmosphere, and then the electrical properties, such as current voltage (I-V), resistance voltage (R-V), and resistance time (R-t), were tested by a self-made test system. The test system consists of an arbitrary waveform generator (Tektronix AWG5002B, Beaverton, OR, USA) and a digital source meter (Keithley-2400, Beaverton, OR, USA). The thin films were continuously annealed at 200–300 °C for 5 min in a N_2_ atmosphere, and the lattice information of the thin films was explored by X-ray diffraction (XRD, Rigaku, Tokyo, Japan).

## 3. Results and Discussion

### 3.1. Atomic Configuration for C-Doped Sb_2_Te_3_

In order to determine the position of the C atom in Sb_2_Te_3_, we first consider four possible doping ways of the C atom in an FCC-Sb_2_Te_3_: replacing the Sb atom (C_Sb_), replacing the Te atom (C_Te_), occupying Sb cation vacancy (C_V_), and interstitial doping (C_I_). On this basis, the formation energy of C doping in an FCC-Sb_2_Te_3_ was calculated and compared. The Equation for calculating the formation energy of C doping is as follows [15,20,21,25]:

(1) $$E^{f}\left[X\right]=E_{\mathrm{tot}}\left[X\right]-E_{\mathrm{tot}}\left[\mathrm{bulk}\right]-\sum_{i}n_{i}\mu_{i},$$

where $E_{\mathrm{tot}}\left[X\right]$ and $E_{\mathrm{tot}}\left[\mathrm{bulk}\right]$ are the total energies of a supercell with and without C doping, respectively, and $n_{i}$ represents the number of doped atoms. $n_{i}>0$ means adding atoms to the supercell, $n_{i}<0$ means removing atoms from the supercell, and $\mu_{i}$ means the chemical potential of i substance. In this paper, the chemical potentials of C, Sb, and Te are calculated according to the simple substance trigonal phase.

**Figure 1:**
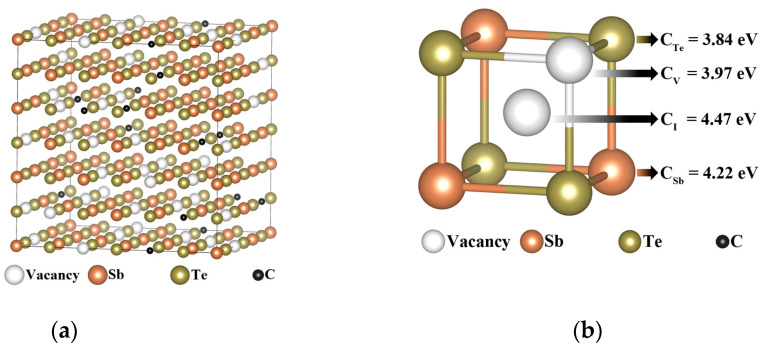
(**a**) The FCC-Sb_2_Te_3_ model with 180 atoms and (**b**) C atoms replace/occupy the formation energies of Sb, Te atoms, Sb vacancies, and intervals in the FCC-Sb_2_Te_3_, respectively.

The FCC-Sb_2_Te_3_ supercell used in the calculation contains 36 cation vacancies, 72 Sb atoms, and 108 Te atoms. The FCC-Sb_2_Te_3_ model is shown in Figure 1a. The calculation results show that the formation energy of C atoms in every position of the FCC-Sb_2_Te_3_ is very high, as shown in Figure 1b, which indicates that the doping system is not easy to form or unstable; that is, the substitution/occupation position of C atoms is unreasonable. 

**Figure 2:**
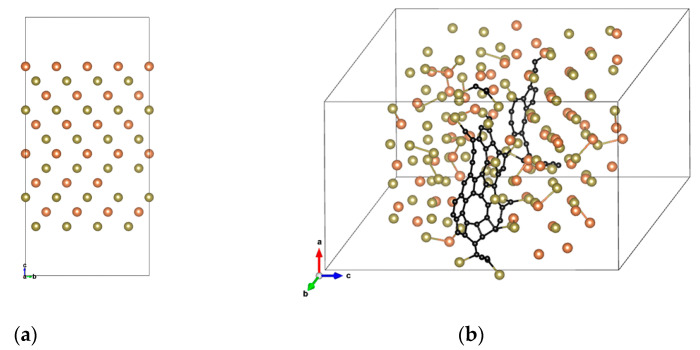
(**a**) The Σ3 twin grain boundary FCC-Sb_2_Te_3_ containing 160 atoms, and (**b**) the C atoms gradually converge at the crystal plane to form C molecular clusters.

It is found from the article [18,25,26,27,28] that C atoms are not simply doped in these four ways, but form C molecular clusters (such as C chain and/or C ring) on the crystal plane. The Σ3 twin grain boundary in an annealed C-Ge_2_Sb_2_Te_5_ alloy accounts for 7.49% of the total polycrystalline structure [28]. Figure 2a shows the FCC-Sb_2_Te_3_ with 160 atoms of Σ3 twin grain boundary, and the thickness of the vacuum layer in the c-axis direction is 10 Å.

**Figure 3:**
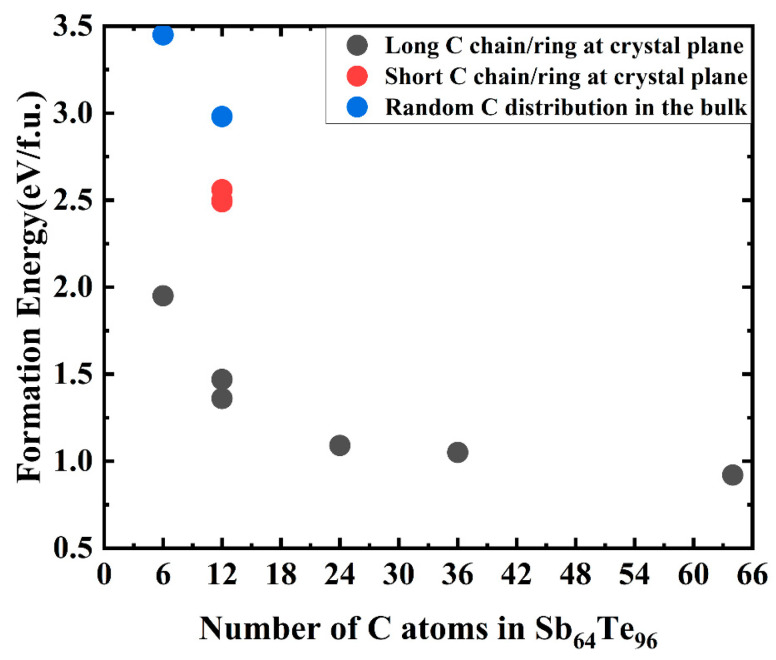
The formation energy of the Sb_2_Te_3_ structure doped with different contents and different forms of C atoms/molecular clusters. The percentages of C atoms are 3.61%, 6.98%, 13.04%, 18.37%, and 28.57%, respectively.

After the structural relaxation, we found that the C atoms have a tendency to gradually converge together and form the C chain and/or C ring [29], as shown in Figure 2b. The longer the C chain, the more C rings, and the lower its formation energy. On the contrary, the more dispersed the C atom is, the higher its formation energy is. Figure 3 shows the formation energies of different C doping contents and forms calculated by Equation (1). As can be seen in Figure 3, comparing the formation energy of a single crystal structure (See Figure 1a) and a twin structure (See Figure 2a), the latter is lower, indicating that C atoms are more inclined to stay at the grain boundary than to replace Sb/Te atoms or occupy intervals/Sb cation vacancies in a single crystal. It is further analyzed that with the increase in C doping concentration, or with the increase in C chain and/or C ring, the formation energy of Sb_2_Te_3_ can gradually decrease, indicating that C atoms tend to converge to each other to form a C chain and/or C ring, which mainly exists in the form of a C chain and/or C ring at the twin grain boundary, as shown in Figure S1. 

**Figure 4:**
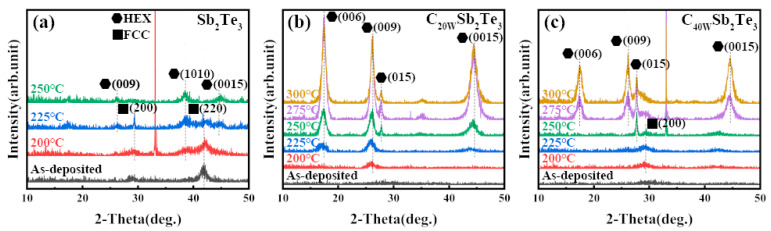
An XRD diagram of Sb_2_Te_3_ and C-Sb_2_Te_3_ in the experiment. (**a**) Sb_2_Te_3_; (**b**) C_20W_Sb_2_Te_3_; and (**c**) C_40W_Sb_2_Te_3_ were annealed for 5 min at different temperatures in a N_2_ atmosphere.

**Figure 5:**
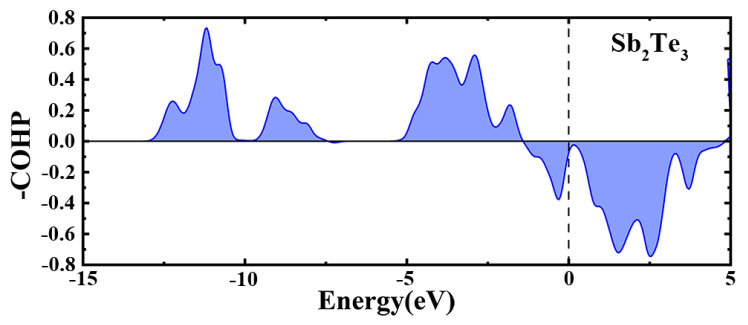
The -COHP curve of Σ3 twin boundary FCC-Sb_2_Te_3_.

It can also be seen in Figure 3 that although the formation energy of C-Sb_2_Te_3_ gradually decreases with the increase in C doping concentration, its value is still greater than 0 (the formation energy of C_64_Sb_64_Te_96_ is 0.92 eV/f.u.), indicating that the structure is not easy to form or unstable. Perhaps this is the reason why the FCC structure was not found in the previous reports of Sb_2_Te_3_ doped with carbon, yet the hexagonal (HEX) structure was found. Yin et al. reported that there was no FCC structure in the experiment of C-doped Sb_2_Te_3_, but the FCC structure was observed in the experiment of N-doped Sb_2_Te_3_ [30] and C-N co-doped Sb_2_Te_3_ [31]. The samples of pure Sb_2_Te_3_ and Sb_2_Te_3_ films doped with different carbon contents were prepared, and their crystal structures were analyzed by XRD, as shown in Figure 4. It can be seen in Figure 4 that when Sb_2_Te_3_ is at 225 °C, the FCC phase and the HEX phase coexist, which indicates that Sb_2_Te_3_ starts to change from the FCC phase to the HEX phase at this time. In C_40W_Sb_2_Te_3_, there is no HEX phase at 200 and 225 °C, but the characteristic peak of the FCC phase appears, and the transition from the FCC phase to the HEX phase begins at 250 °C. However, the characteristic peak of the FCC phase was not observed at 225–250 °C in C_20W_Sb_2_Te_3_, which indicated that with the increase in C doping concentration, the formation energy of C-Sb_2_Te_3_ decreased, so that the FCC phase can appear in C-doped Sb_2_Te_3_, and the FCC phase is likely to be stable in the C-Sb_2_Te_3_ structure as the C concentration increases. The formation energy of the metastable FCC-Sb_2_Te_3_ structure constructed by us is calculated to be 0.04eV/f.u., which is close to kT = 0.026 eV at room temperature. However, the formation energy of the FCC-Sb_2_Te_3_ structure is greater than 0, which also indicates that its stability is poor. In order to further understand the chemical stability of the Sb_2_Te_3_ structure, we performed the COHP analysis for Sb_2_Te_3_, as shown in Figure 5. The upper and lower portions of the -COHP curve indicate bonding (stable) and anti-bonding (unstable) interactions, respectively. From the -COHP of Sb_2_Te_3_ in Figure 5, the existence of the anti-bonding state of Sb-Te atoms below Fermi level (E_f_) also indicates that the stability of the FCC-Sb_2_Te_3_ structure is poor [32], where the cutoff distance of an Sb-Te bond is 3.1 Å [18,28,33,34]. Kolobov et al. theoretically predicted and constructed the structure model of the FCC-Sb_2_Te_3_ in 2013 [35], but it was not until 2016 that Zheng et al. observed the structure of the FCC-Sb_2_Te_3_ in the experiment for the first time using TEM analysis [34].

### 3.2. Electronic Properties and Origin of Change of Crystalline C-doped Sb_2_Te_3_

**Figure 6:**
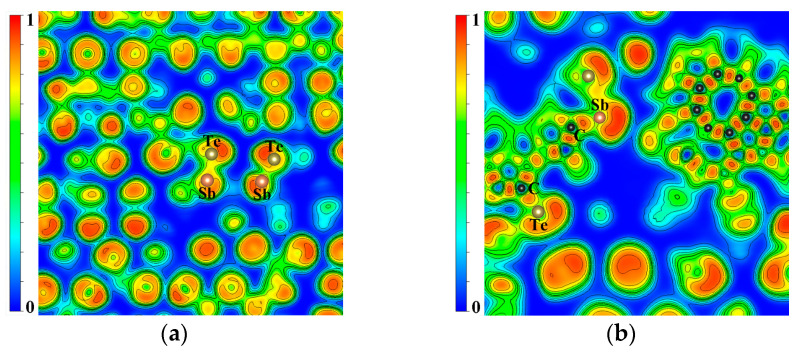
Two-dimensional electronic local function contour plots of Sb_2_Te_3_ and C-Sb_2_Te_3_. (**a**) Σ3 twin grain boundary FCC-Sb_2_Te_3_ without doping C atoms and (**b**) Σ3 twin grain boundary FCC-Sb_2_Te_3_-doped C atoms.

To understand the mechanism of the thermal stability improvement of C-doped Sb_2_Te_3_, contour plots of electron localization function (ELF) projected on the same planes for Sb_2_Te_3_ and 64C-Sb_2_Te_3_ are shown in Figure 6, and the ELF maxima of various bonds are shown in Table 1. It is shown that the ELF maximum of the C-C bond is much higher than that of other bonds, indicating that the strength of the C-C bond is very high, which also proves that C atoms mainly exist in the form of C molecular clusters in Sb_2_Te_3_. The ELF maximum values of C-Te and C-Sb bonds are obviously much higher than 0.5, which indicates that C-Te and C-Sb bonds have high strength; that is, there are some molecular clusters containing C-Te and C-Sb. In addition, it also shows that the doping of the C atoms changes the local environment of each element in Sb_2_Te_3_ and increases the strength of the Sb-Te covalent bond, thus obviously improving the stability of Sb_2_Te_3_ after C doping [36,37]. 

**Figure 7:**
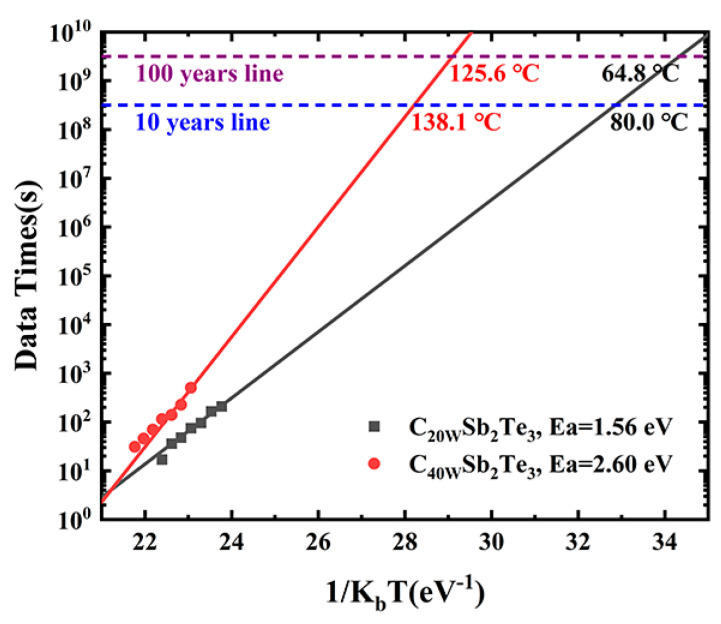
The 10-year (or 100-year) data retention temperature and activation energy of crystallization are deduced based on the Arrhenius equation, according to the failure time versus reciprocal temperature.

In order to represent the effect of C element doping on the amorphous thermal stability of Sb_2_Te_3_ materials, we tested the failure time of thin film materials at different temperatures. The failure time is considered to be that the film resistance drops to half of the initial resistance at this set temperature T. As shown in Figure 7, the 10-year (and 100-year) data retention is estimated according to the Arrhenius equation:

(2) $$t=\tau \exp\left(\frac{E_{a}}{k_{b}T}\right),$$

where t is the failure time of the film at a set temperature T, τ is the preexponential factor, E_a_ is the activation energy, and K_b_ is the Boltzmann constant. It can be seen in Figure 7 that the addition of C atoms obviously improves the 10-year (or 100-year) data retention of Sb_2_Te_3_.

**Figure 8:**
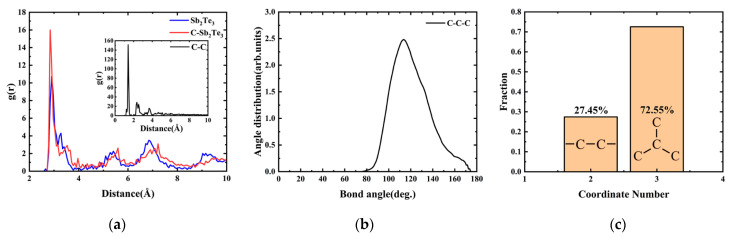
(**a**) Pair correlation function (PCF) of the Sb-Te bond and the C-C bond in crystal Sb_2_Te_3_ and C-Sb_2_Te_3_ after structural relaxation; (**b**) bond angle distribution of the C-C-C configuration; and (**c**) coordination number statistics of C atoms.

Figure 8a shows the pair correlation function (PCF) of the C-C bond and the Sb-Te bond in crystal Sb_2_Te_3_ and C-Sb_2_Te_3_ after structural relaxation. For the first peak, we found that the peak value of the C-C bond is far greater than that of the Sb-Te bond, C-Sb bond, C-Te bond, etc., indicating that C atoms are more inclined to combine with C atoms in Sb_2_Te_3_ to form C molecular clusters. To our surprise, the position of the first peak of the C-C bond is 1.406 Å, which is very close to the inter-layer atomic spacing of 1.42 Å in graphite structure [38,39]. In addition, the maximum bond angle distribution of the C-C-C configuration in C molecular clusters is about 105–125°, and the coordination number of C atoms is mainly 3-coordinate, as shown in Figure 8b,c. These indicate that the doped C atoms in Sb_2_Te_3_ are not randomly formed when forming C molecular clusters, but tend to form graphite-like layered structures by sp^2^ hybridization [18,28]. The low PCF peaks of the C-Sb and C-Te bonds in Figure S2 indicate that C atoms bond less with Sb and Te atoms. It is observed that after doping the C atom in Sb_2_Te_3_, the position of the first peak value of the Sb-Te bond decreases and the bond length becomes shorter, indicating that the binding between Sb and Te atoms is strengthened, thus making the structure more stable. Meanwhile, the extremely unstable Sb-Sb homobonds are reduced, which also contributes to the enhancement of structural stability, as shown in Figure S2.

### 3.3. Electrical Performance Test of a Prototype PCRAM Device Based on C-Sb_2_Te_3_ Material

**Figure 9:**
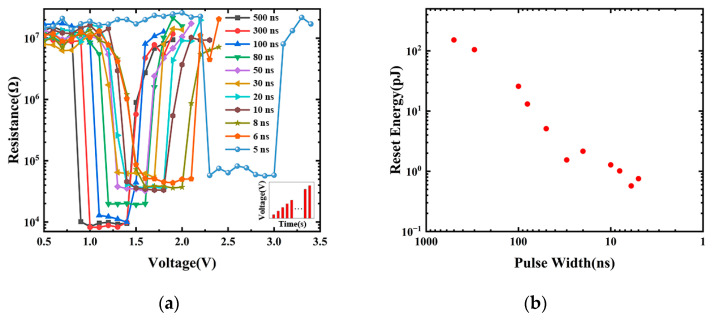
(**a**) The R-V electrical test of the prototype PCRAM device based on C-Sb_2_Te_3_ material, and (**b**) the relationship between power consumption and pulse width.

**Figure 10:**
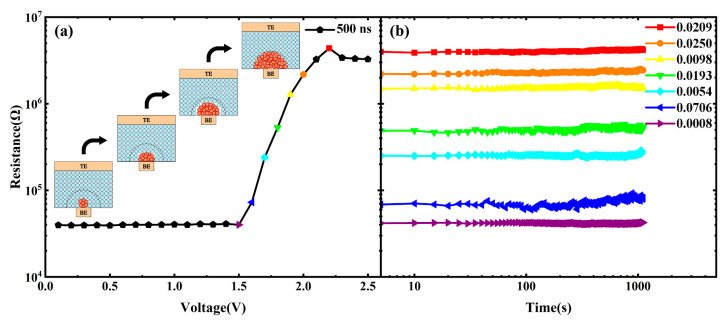
(**a**) The R-V curve is obtained by resetting the voltage pulse with a pulse width of 500 ns, and (**b**) the R-t curve and the resistance drift coefficient ν is obtained by fitting with Equation (2).

The electrical programming characteristics of the C-Sb_2_Te_3_ prototype PCRAM device are shown in Figure 9a. The illustration is the voltage pulse we applied to the PCRAM cell and the pulse width is fixed and the voltage amplitude step is set to 0.1 V. Starting from the first pulse amplitude of 0.1 V until the PCRAM cell stops after the SET and the RESET, as illustrated in Figure 9a, a reading voltage of 0.1 V is applied between every two pulses to record the resistance value of the PCRAM test cell. The initial state resistance value and the final state resistance value are controlled to be equal as much as possible. Obviously, the final state resistance value before adjusting the voltage pulse width is taken as the initial state resistance value after adjusting the voltage pulse width, and the initial state resistance value will affect the SET voltage value and even the RESET voltage value of this electrical programming. It can be seen from Figure 9a that the resistance resolution of the PCRAM cells exceeds two orders of magnitude, which meets the requirements of PCRAM. Our PCRAM device cells can be programmed at a very low SET/RESET voltage with a pulse width of 500 ns–5 ns, and the SET voltage and RESET voltage are as low as 1.5 V and 2.2 V when the voltage pulse width is 6 ns, which indicates that our PCRAM device has an operating speed of 5 ns and a low device power consumption (0.57 pJ), as shown in Figure 9b. Further observation in Figure 9b shows that the RESET power consumption of the PCRAM device cell decreases with the decrease in the width of the programming pulse. At the same time, it was noted that the PCRAM device cell could not be completely SET operated, as the width of the programming pulse decreased, resulting in the resistance value of its low resistance state to increase. This explains the behavior of the RESET power consumption change of the PCRAM device cell. However, the PCRAM device cell cannot be completely SET, which may be caused by the FCC phase appearing before the amorphous phase is transformed into the HEX phase due to the high C doping concentration. The multistage storage function can be realized by setting different voltage pulse widths and voltage pulse amplitudes, such as “0” at 10^7^ Ω, “1” at 10^5^ Ω, and “2” at 10^4^ Ω [40]. We also noticed that during the SET/RESET process of our PCRAM device, there was a continuous resistance change. In this case, we used a voltage pulse with a pulse width of 500 ns to RESET the PCRAM in a low resistance state, and the continuously adjustable resistance value can be obtained as shown in Figure 10a. With this change, maybe we can realize several basic synaptic functions at the cell level, including long-term plasticity (LTP) [41,42], short-term plasticity (STP) [41,42], spike timing-dependent plasticity (STDP) [43,44], and spike rate-dependent plasticity (SRDP) [44,45], and maybe can also realize more complex or higher-order learning behaviors at the network level, such as supervised learning [46] and associative learning [47], as well as non-von Neumann architecture of in-memory computing [48,49]. In general, for this phenomenon of continuous resistance change, the resistance drift caused by the widening of the band gap due to the structural relaxation (SR) of amorphous Sb_2_Te_3_ is a great obstacle to multilevel storage, neuromorphic learning and in-memory computing. Li et al. greatly reduced this resistance drift phenomenon by bipolar pulse operation on the PCRAM cell [50], which provides an effective means to improve the stability of the phase-change neuromorphic applications. We adjusted the resistance values of the PCRAM cell to high and low resistance states and each intermediate resistance state by controlling the electrical signal applied to the PCRAM cell, and fitting the resistance drift coefficient by Equation (3):

(3) $$R\left(t\right)=R_{0}{\left(\frac{t}{t_{0}}\right)}^{\nu },$$

where *R*_0_ is the resistance value of the PCRAM cell at time t_0_; that is, the initial resistance. ν is the resistance drift coefficient, indicating the change in the resistance value of the PCRAM cell with time. The results measured at room temperature are shown in Figure 10b.

## 4. Conclusions

We have obtained an understanding of the doping position and existence form of C atoms in the FCC-Sb_2_Te_3_ and the improved device performance after C doping by performing density functional theory calculations for different concentrations and forms of C doping in single crystal FCC-Sb_2_Te_3_ and Σ3 twin boundary FCC-Sb_2_Te_3_. The results show that the formation energy of C-Sb_2_Te_3_ decreases with the increase in C doping concentration, which is consistent with the appearance of the FCC phase in high-concentration C-doped Sb_2_Te_3_ in our experiment. In addition, C atoms prefer to form C molecule clusters by sp^2^ hybridization at the grain boundary of Sb_2_Te_3_, similar to the layered structure of graphite, which changes the local environment of each element in Sb_2_Te_3_, resulting in the improvement of thermal stability of Sb_2_Te_3_. We fabricated the prototype device cells of PCRAM, which had an operating speed of 5 ns, a high thermal stability (10-year data retention temperature 138.1 °C), a low device power consumption of 0.57 pJ, and a resistance drift coefficient as low as 0.025, showing a continuously adjustable resistance. These performances all indicate that C-Sb_2_Te_3_-based PCRAM devices have great potential in applications, such as multilevel storage and spiking neural networks.

## Figures and Tables

**Table 1:** The maximum electronic local function value of each covalent bond in the FCC-Sb_2_Te_3_ with Σ3 twin grain boundary of undoped/doped C atoms in the direction of bond length.

Bonds	Sb_2_Te_3_	C-Sb_2_Te_3_
Sb-Te	0.78	0.80
C-C	/	0.94
C-Sb	/	0.91
C-Te	/	0.89

## Data Availability

Not applicable.

## Supplementary Materials

The following supporting information can be downloaded at https://www.mdpi.com/article/10.3390/nano13040671/s1, Figure S1: After structural relaxation of different C doping contents and C existing forms; Figure S2: The pair correlation function after structure relaxation; Figure S3: XRD diagram of C_40W_Sb_2_Te_3_ at 225 °C; Figure S4: The optical image of the fabricated PCRAM cells; Figure S5: I-V curve of PCRAM unit set from high resistance state to low resistance state by DC current; Figure S6: SET the PCRAM in a low resistance state with a large/small set pulse, and then RESET it with a 500 ns pulse to obtain a continuously adjustable resistance value.
