# Supplementary material for: The Effect of Carbon Doping on the Crystal Structure and Electrical Properties of Sb2Te3
Source: Nanomaterials (Basel). 2023 Feb 9;13(4):671. doi: 10.3390/nano13040671 (PMC9959287; doi:10.3390/nano13040671)
Supplement: Supplementary file 1 [file nanomaterials-13-00671-s001.zip › nanomaterials-2154918-supplementary.pdf]

Supplementary Materials

The Effect of Carbon Doping on the Crystal Structure and Electrical Properties of Sb_2Te_3

Jie Zhang [1], Ningning Rong [1], Peng Xu [1], Yuchen Xiao [1], Aijiang Lu [1], Wenxiong Song [2], Sannian Song [2], Zhitang Song [2], Yongcheng Liang [1] and Liangcai Wu [1,3,*]

[1] College of Science, Donghua University, Shanghai 201620, China

[2] State Key Laboratory of Functional Materials for Informatics, Shanghai Institute of Microsystem and Information Technology, Chinese Academy of Sciences, Shanghai 200050, China

[3] Shanghai Institute of Intelligent Electronics & Systems, Shanghai, China

* Correspondence:

Corresponding Author

lcwu@dhu.edu.cn (L.W.)

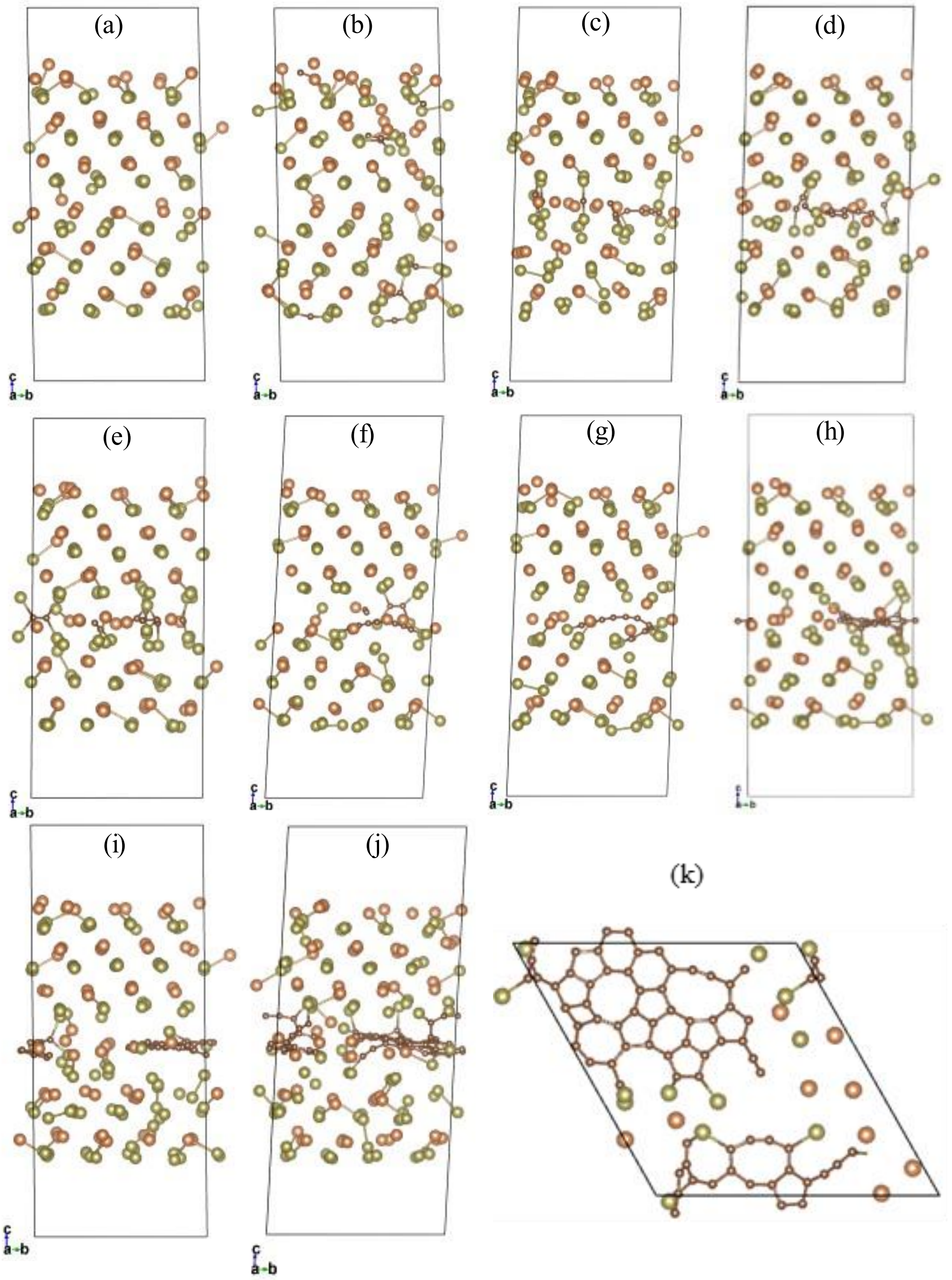

Figure S1. After structural relaxation of different C doping contents and C existing forms. (a) Sb_2Te_3, (b) 12 C atoms are randomly distributed in Sb_2Te_3, (c) 12 C atoms are randomly distributed in the crystal plane, (d) (e) the short C chains (12 C atoms in

total) is distributed in the crystal plane, (f) (g) the long C chain (12 C atoms in total) is distributed in the crystal plane, (h) the long C chain (24 C atoms in total) is distributed in the crystal plane, (i) the long C chain (36 C atoms in total) is distributed in the crystal plane, (j) the long C chain (64 C atoms in total) is distributed in the crystal plane, (k) Top view of C atoms distribution in figure (j). Note that the initial positions of C atoms in Figures (b) and (c) are scattered, and after the structure relaxes, the C atoms gradually converge to form a short C chain.

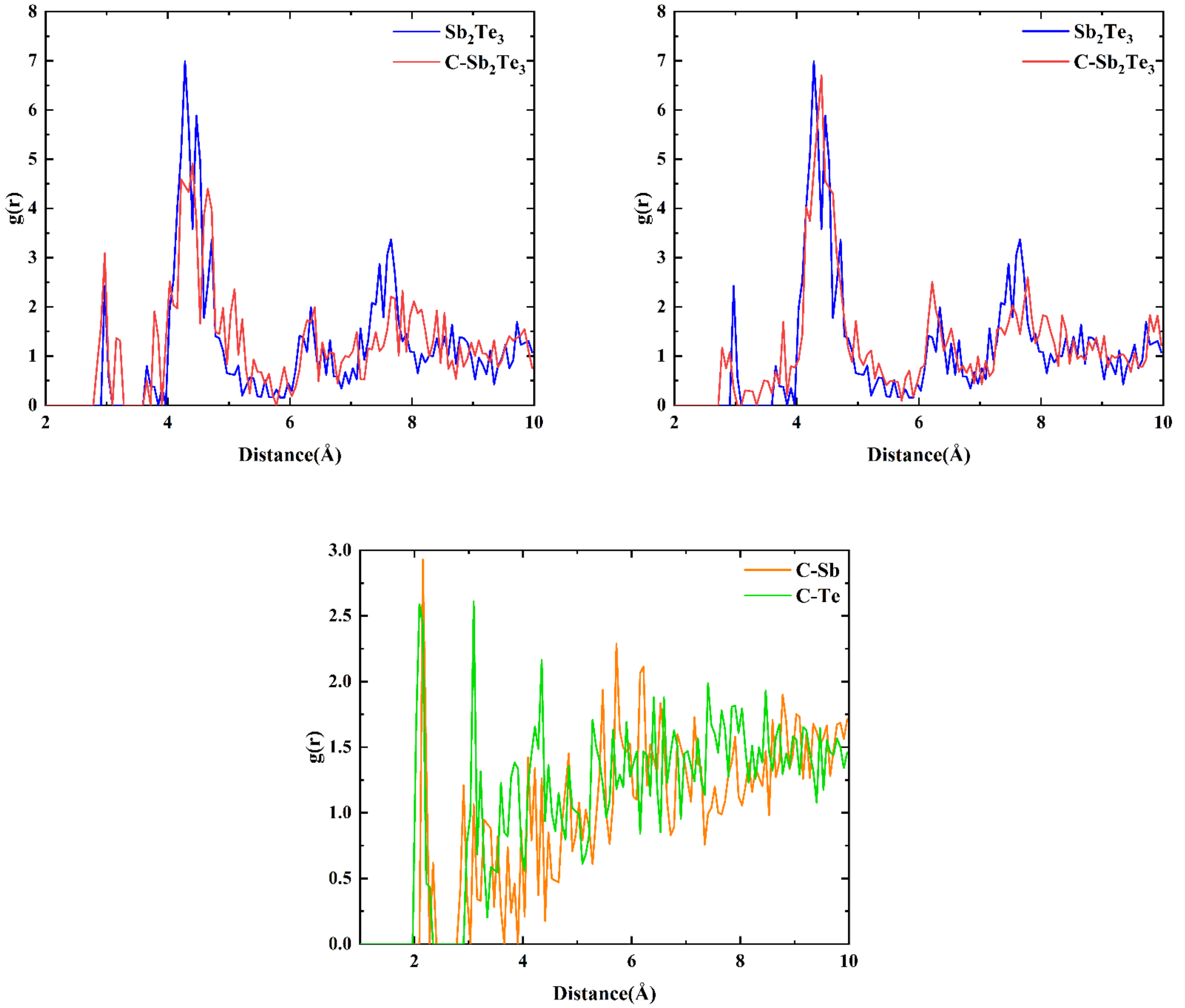

Figure S2. The pair correlation function after structure relaxation. (a)Sb-Sb bond, (b)Te-Te bond, (c)C-Sb bond and C-Te bond.

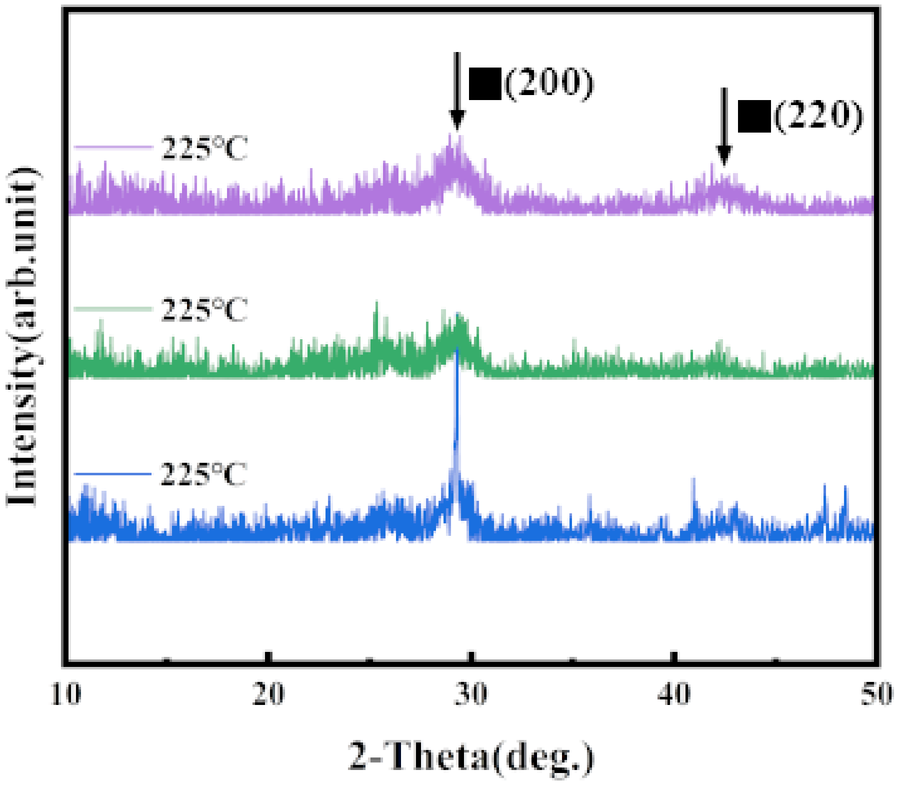

Figure S3. XRD diagram of $C_{40}WSb_2Te_3$ at 225°C.

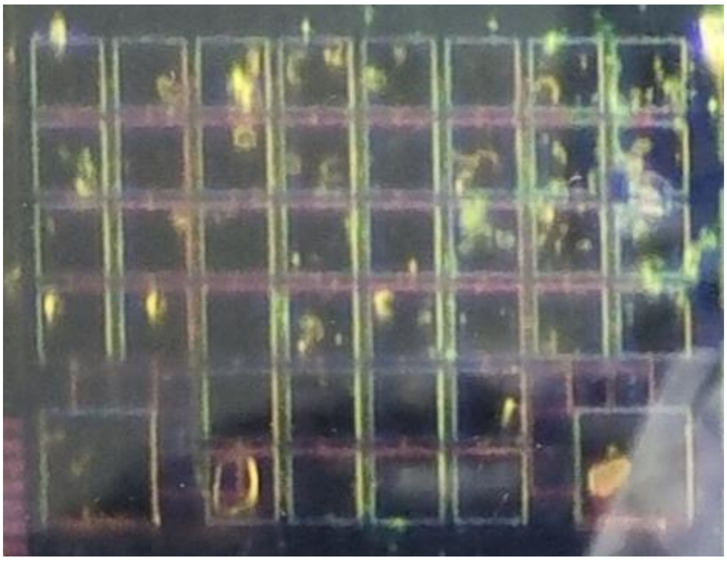

Figure S4. The optical image of the fabricated PCRAM cells.

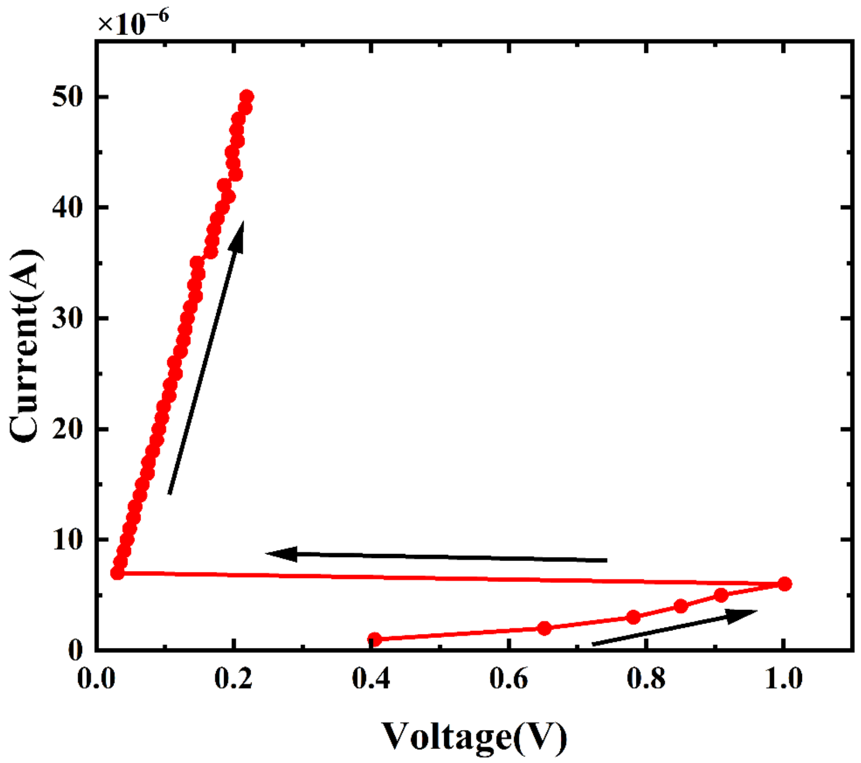

Figure S5. I-V curve of PCRAM unit set from high resistance state to low resistance state by DC current.

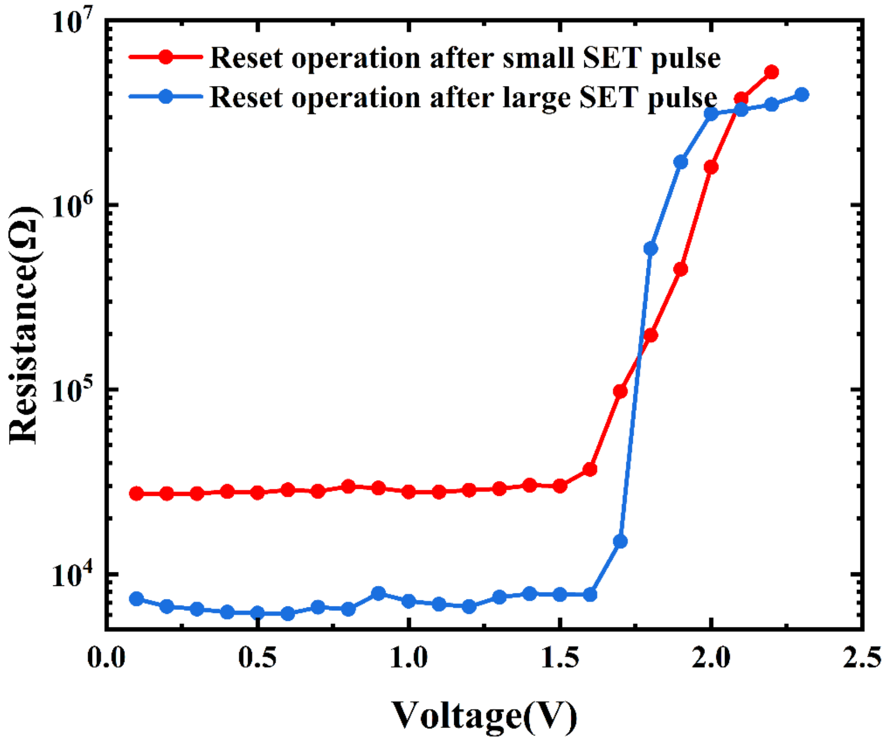

Figure S6. SET the PCRAM in a low resistance state with a large / small set pulse, and then RESET it with a 500ns pulse to obtain a continuously adjustable resistance value.
